# Artesunate-modified nano-graphene oxide for chemo-photothermal cancer therapy

**DOI:** 10.18632/oncotarget.21191

**Published:** 2017-09-23

**Authors:** Yilin Pang, Zihao Mai, Bin Wang, Lu Wang, Liping Wu, Xiaoping Wang, Tongsheng Chen

**Affiliations:** ^1^ MOE Key Laboratory of Laser Life Science & College of Biophotonics, South China Normal University, Guangzhou 510631, PR China; ^2^ Department of Pain Management, The First Affiliated Hospital of Jinan University, Guangzhou 510630, PR China

**Keywords:** artesunate, nano-graphene oxide, chemo-photothermal synergistic therapy, peroxynitrite, near-infrared irradiation

## Abstract

Poor water-solubility of artesunate (ARS) hampers its clinical application. We here covalently linked ARS to PEGylated nanographene oxide (nGO-PEG) to obtain ARS-modified nGO-PEG (nGO-PEG-ARS) with excellent photothermal effect and dispersibility in physiological environment. nGO-PEG-ARS induced reactive oxygen species (ROS) and peroxynitrite (ONOO─) generations. Although nGO-PEG with near-infrared (NIR) irradiation did not induce cytotoxicity, the photothermal effect of nGO-PEG under NIR irradiation enhanced not only cell uptake but also ONOO─ generation of nGO-PEG-ARS, resulting in the synergistic chemo-photothermal effect of nGO-PEG-ARS in killing HepG2 cells. Pretreatment with Fe(III) 5,10,15,20-tetrakis (4-sulfonatophenyl) porphyrinato chloride (FeTTPS, a ONOO─ scavenger) instead of antioxidant N-Acetyle-Cysteine (NAC, an ROS scavenger) significantly blocked the cytotoxicity of nGO-PEG-ARS with or without NIR irradiation, demonstrating that ONOO─ instead of ROS dominated the synergistic chemo-photothermal anti-cancer action of nGO-PEG-ARS. nGO-PEG-ARS with NIR irradiation resulted in a complete tumor cure within 15 days earlier than other treatment groups, and did not induce apparent histological lesion for the mice treated with nGO-PEG-ARS with or without NIR irradiation for 30 days, further proving the synergistic chemo-photothermal anti-cancer effect of nGO-PEG-ARS. Collectively, nGO-PEG-ARS is a versatile nano-platform for multi-modal synergistic cancer therapy.

## INTRODUCTION

Nanoparticle therapeutics has been demonstrated to be a potential multi-modal approach to enhance efficacy and simultaneously reduce side effects of cancer treatment [1–3]. Physical and psychosocial side effects in patients receiving traditional cancer therapies including surgery, radiotherapy, chemotherapy, hormonal therapy and immunotherapy have led to an inexorable trend towards devising more effective therapy in a wide-ranging manner for cancers [4, 5]. The properties of nanoparticles such as more targeted localization in tumors and active cellular uptake make it possible to achieve controlled release drug delivery and specific gene transfection [2, 6, 7]. Photothermal sensitivity of nanoparticles can be used for photothermal therapy (PTT) and photodynamic therapy (PDT) *in vitro* and *in vivo* [8–11]. Moreover, multiple-modal therapeutic ability of nanoparticles significantly improves the therapeutic efficiency of cancer treatment [12–16].

Nano-graphene oxide (nGO) is a potential nano-platform for chemo-photothermal synergistic therapy [17–20]. High photothermal responsiveness and low toxicity make nGO a promising photo-absorbing agent for PTT [16, 21, 22]. nGO also has ultrahigh loading capacity of aromatic drugs due to its large specific surface area and the strong non-covalent binding (hydrophobic interactions and *π*-*π* stacking) between aromatic molecules and aromatic regions of nGO [23–25]. Yang and colleagues reported that nGO loaded doxorubicin (DOX) as high as 235% (the weight ratio of loaded drug to carriers) by *π*-*π* stacking and hydrophobic interactions [26]. Liu and colleagues conjugated six-armed PEG-amine stars to the carboxylic acid groups on GO via carbodiimide-catalyzed amide formation to form PEGylated nGO (nGO-PEG) loading about 10% SN38 (a water insoluble drug) by noncovalent binding [27]. Zhang and colleagues developed DOX-loaded nGO-PEG to combine chemotherapy and PTT in one system, which significantly improved the therapeutic efficacy of mammary cancer treatment in *in vivo* and *in vitro* [17]. We recently synthetized noncovalently PEGylated nrGO (nrGO/PEG) loading about 175.6 w/w % of resveratrol (RV) via *π*-*π* and hydrophobic interactions and exhibiting dramatically synergistic anti-cancer effect of PTT and chemotherapy *in vitro* and *in vivo* [28].

Artesunate (ARS) and dihydroartemisin (DHA), the first line anti-malarial drugs with only minimal side-effects, possess profound anticancer activity against various cancer cell lines [29–31]. However, poor water solubility and low bioavailability of ARS and DHA limits their clinical applications [30, 32, 33]. Polyethylene glycol (PEG), a modifying polymer, is widely used to increase the solubility and sustained release of macromolecular drug [34, 35]. Chadha and colleagues mixed PEG and β-cyclodextrin ARS in water to significantly improve the solubilizing efficiency and bioavailability as well as antimalarial activity of ARS [36]. Dai and colleagues linked DHA to carboxylic-terminated PEG through covalent bonds between the carboxylic groups of PEG and hydroxide radicals of DHA to synthetize PEGylated DHA (PEG-DHA) that was significantly more effective than DHA in the lewis lung carcinoma xenograft model [37].

**Scheme 1:**
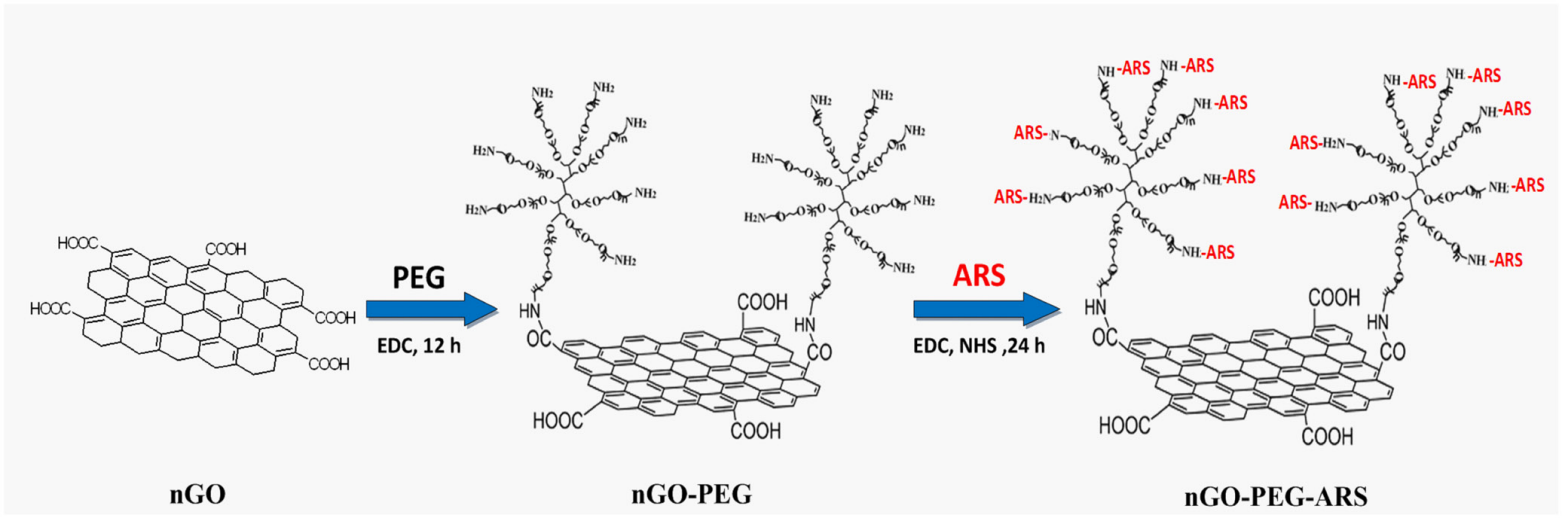
Schematic illustration of the synthetic procedure of GO-PEG-ARS (Note: the scales used in this reaction schematic do not denote the real situation)

In this report, we developed an ARS-loaded nGO-PEG composite (nGO-PEG-ARS) for synergistic chemo-photothermal therapy of cancer via two steps (Scheme 1): 1) covalent PEGylation of nGO (nGO-PEG); 2) loading ARS onto nGO-PEG through covalent binding between the carboxylic groups of and the amino groups of nGO-PEG. Near-infrared laser (NIR) irradiation significantly enhanced the cytotoxicity of nGO-PEG-ARS, while nGO-PEG had no noticeable toxicity in human hepatocellular carcinoma cells (HepG2) regardless of NIR irradiation. Interestingly, Peroxynitrite (ONOO^**─**^) not reactive oxygen species (ROS) dominated the synergistic chemo-photothermal anti-cancer effect of nGO-PEG-ARS. In addition, the mild photothermal effect of nGO-PEG by NIR irradiation significantly enhanced cell uptake and ONOO^**─**^ generation of nGO-PEG-ARS, contributing to the outstanding synergistic chemo-photothermal anti-cancer effect of nGO-PEG-ARS, which was verified by *in vivo* experiments in Balb/c mice bearing 4T1 murine breast cancer tumors.

## RESULTS AND DISCUSSION

### Characterization of nGO-PEG-ARS

**Figure 1:**
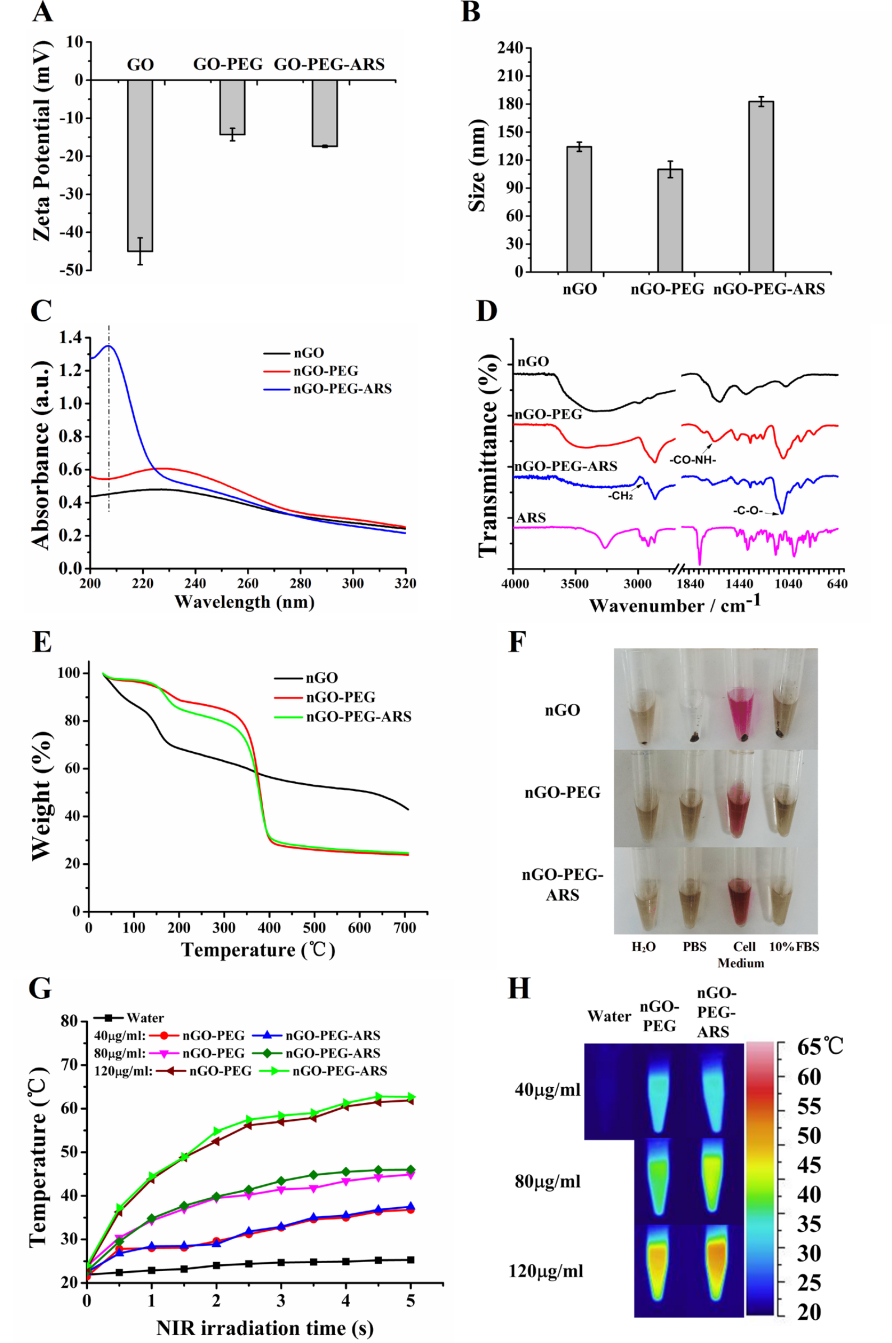
Characterizations of GO-PEG-ARS **(A-F)** Zeta potential (A), Size (B), FT-IR spectra (C), UV−vis spectra (D), TGA (E) and Dispersity in different solutions after centrifuged at 4800 × g for 10 min (F) of nGO, nGO-PEG and nGO-PEG-ARS. **(G)** Temperature curves versus time during irradiation with 808 nm laser (2 W/cm^2^) for vials containing water, nGO, nGO-PEG, and nGO-PEG-ARS solutions with 40, 80 and 120 μg/mL of GO. **(H)** Thermal images of vials containing water, nGO, nGO-PEG, and nrGO-PEG/PEI solutions with 40, 80 and 120 μg/mL of GO after irradiation for 5 min by 808 nm laser irradiation.

Zeta potential analysis was used to verify the modifications of both ARS and PEG onto nGO. nGO-PEG had a decreased negative potential (-14.8 mV) compared with nGO (-45.4 mV) due to the reaction between the negative carboxyl groups of nGO and the NH_2_ groups of PEG, whereas the zeta potential of nGO-PEG-ARS increased to -17.6 mV due to the linkage of ARS (Figure 1A). As shown in Figure 1B, the size of nGO, nGO-PEG, GO-PEG-ARS was about 134, 110, and 182 nm respectively, indicating that PEGylation reaction decreased the size of nGO and ARS was successfully connected to nGO-PEG.

The UV-Vis spectra of nGO, nGO-PEG and nGO-PEG-ARS solutions were exhibited in Figure 1C. Covalent PEGylation did not significantly influence the NIR absorbance property of nGO, but nGO-PEG had a higher optical absorption in the 2000-2100nm than nGO, indicating the successful covalent reaction between nGO and PEG. Because of low absorption at low wavelengths and relatively low molar extinction coefficient as well as no distinct UV/vis spectra or fluorescent properties, ARS is particularly difficult to be detected and identified by standard spectrophotometric methods [38]. The new absorption peak (∼208 nm) of nGO-PEG-ARS (Figure 1C) demonstrated successful the formation of nGO-PEG-ARS conjugates. FTIR spectrum was used to further confirm the successful synthesis of nGO-PEG-ARS. In the FT-IR spectrum, most absorption peaks of OH (∼3425 cm^-1^), C=O (∼1726 cm^-1^), C=C (∼1575 cm^-1^) and C-O (∼1059 cm^-1^) are the functional groups of the carboxylated nGO [39, 40], and obvious CH2 (∼2900 cm^-1^) and C-O-C (∼1100 cm^-1^) stretching vibrations are the functional groups of PEG [41], which also appeared in the FTIR spectrum of nGO-PEG with only a slight shift of peak positions and change of relative intensity (Figure 1D), indicating the successful synthesis of nGO-PEG. Moreover, the new stretching vibration presented at ∼2900 cm^-1^ (-CH2) and strong stretching vibration presented at ∼1100 cm^-1^ (C-O-C) (Figure 1D) demonstrated the successful synthesis of nGO-PEG-ARS [41]. We next performed TGA for nGO, nGO-PEG and nGO-PEG-ARS to characterize the binding of ARS and PEG onto nGO. As shown in Figure 1E, nGO group continuously lose weight from ambient temperature to 130 °C, which resulted from the loss of adsorbed water molecules covering the nGO sheets [42]. nGO-PEG group showed a first decomposition of nGO in the range from 160 °C to 230 °C and a second large weight loss (about 50%) with an onset temperature at 330 °C due to the thermal decomposition of PEG [42]. Moreover, the weight loss of 21 % of nGO-PEG-ARS was more than the weight loss of 13 % of nGO-PEG in the range from 130 °C-330 °C (Figure 1E), which might be due to the decomposition of ARS that decomposes at 152.8 °C of threshold temperature [43]. All these data corroborated that both PEG and ARS were covalently connected onto the surface of nGO.

### Biocompatibility and photothermal effect

PEGylation was designed to impart the high degree of hydrophilicity for nGO [17]. In order to confirm the dispersibility of GO materials in aqueous solution, we evaluated the dispersion of these nGO materials in water, PBS, 10% FBS and cell medium (DMEM containing 10 % of FBS) for 2 weeks, and found that after centrifuging at 4800g for 10 min, nGO-PEG and nGO-PEG-ARS (n50 μg/mL GO) were very stable in these solvents, while nGO was aggregated in PBS solution and cell medium at room temperature (Figure 1F), indicating the successful conjunction of PEG. The excellent water-solubility and stability in physiological solutions make nGO-PEG an excellent drug-carrier platform.

Excellent water-solubility of nGO-PEG-ARS can not only retain the biological activity of ARS but also effectively reduce the dose of intravenous injections due to the potentially prolonged duration of nGO-PEG-ARS [44, 45]. nGO-PEG-ARS with appropriate nanosize, can migrate through open malignant neovasculature and also increase drug residence time in tumor tissues via the enhanced permeability and retention (EPR) effect [37]. After intravenous injection, nGO-PEG-ARS may result in a prolonged circulation time of ARS, and thus decrease constant repeated administration of drugs and reduce the possibility of emerging ARS resistance in cancer cells during chemotherapy. In addition, PEGylated GO is also used as an excellent photothermal agents for quick temperature rising under NIR irradiation and the synergistically enhanced anti-cancer effect of drugs [16, 21].

To verify the potential of nGO-PEG-ARS as a photothermal agent, nGO-PEG and nGO-PEG-ARS (40, 80 and 120 μg/mL of nGO) were exposed to the 808 nm NIR laser at a power density of 2 W/cm^2^ for 5 min. After NIR irradiation for 5 min, control water had no response to NIR irradiation, whereas the temperature of nGO-PEG/nGO-PEG-ARS solutions increased to ∼35 °C (40 μg/mL), 44 °C (80 μg/mL) and 60 °C (120 μg/mL) (Figure 1G), which was verified by thermal images of nGO-PEG/nGO-PEG-ARS solutions (40-120 μg/mL) (Figure 1H).

### Photothermally enhanced delivery of nGO-PEG-ARS

**Figure 2:**
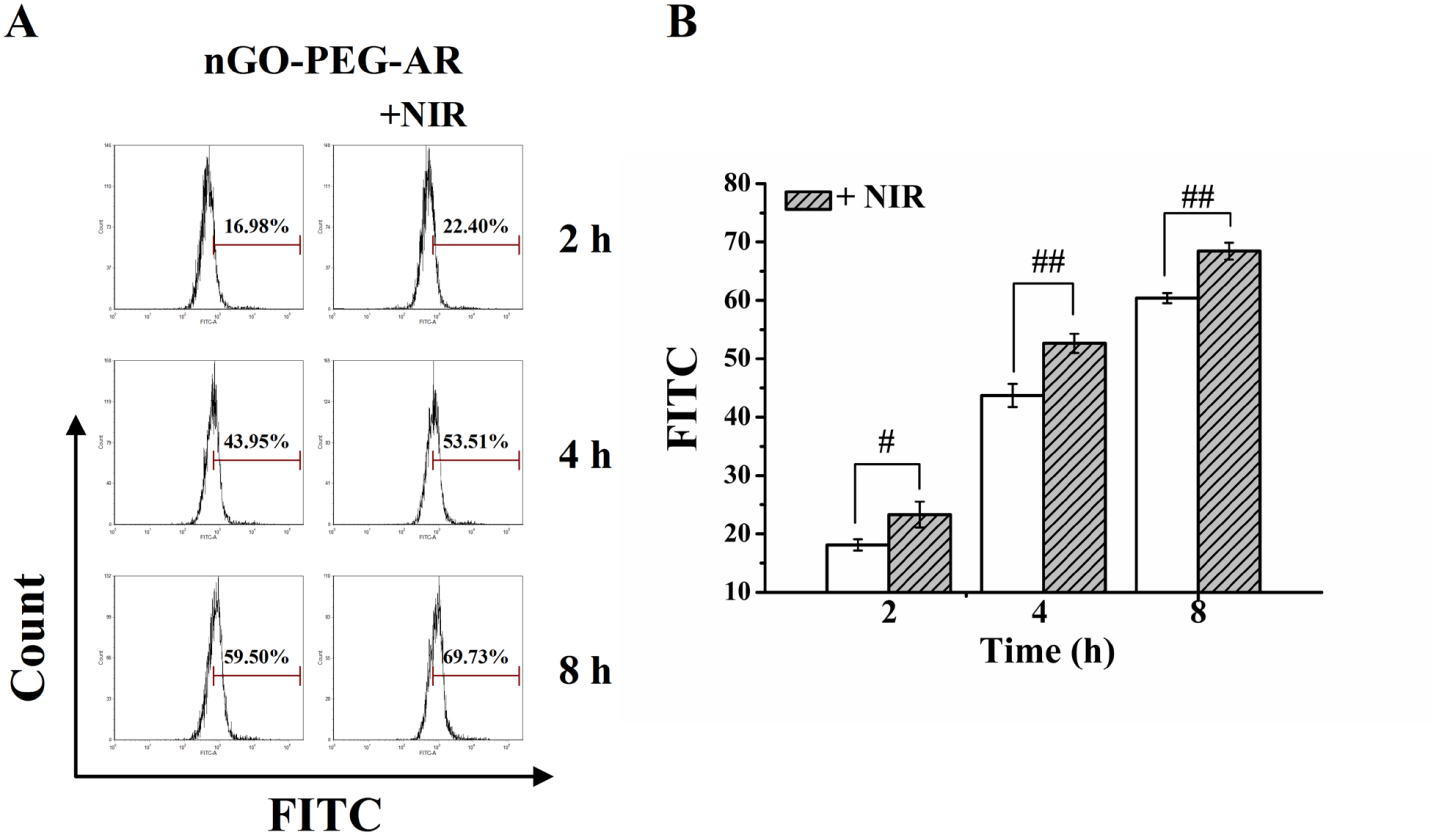
Photothermally enhanced delivery of nGO-PEG-ARS **(A)** Cell uptake of nGO-PEG-ARS/FITC in the absence or presence of NIR irradiation analyzed by FCM analysis. **(B)** Statistical results of cell uptake of nGO-PEG-ARS/FITC in the absence or presence of NIR irradiation from three independent experiments. ^**##**^*P* < 0.01 and ^**###**^*P* < 0.001.

It has been demonstrated that mild photothermal heating to ∼43°C of nanoparticles by lower power NIR laser irradiation was able to enhance the intracellular delivery of chemotherapy drugs or photosensitizers for improved cancer cell killing [46, 47]. To verify the enhanced intracellular trafficking of nGO-PEG-ARS after NIR irradiation, we assessed the cell uptake of FITC-labeled nGO-PEG-ARS at different incubation time (2, 4 and 8 h) in the presence or absence of NIR irradiation by using FCM analysis. FCM analysis (Figures 2A and 2B) showed that NIR irradiation significantly enhanced the cellular uptake of nGO-PEG-ARS, which may be owing to the increased cell membrane permeability at a slightly higher temperature [46].

### *In vitro* synergistic chemo-photothermal anti-cancer effect of nGO-PEG-ARS in HepG2 cells

**Figure 3:**
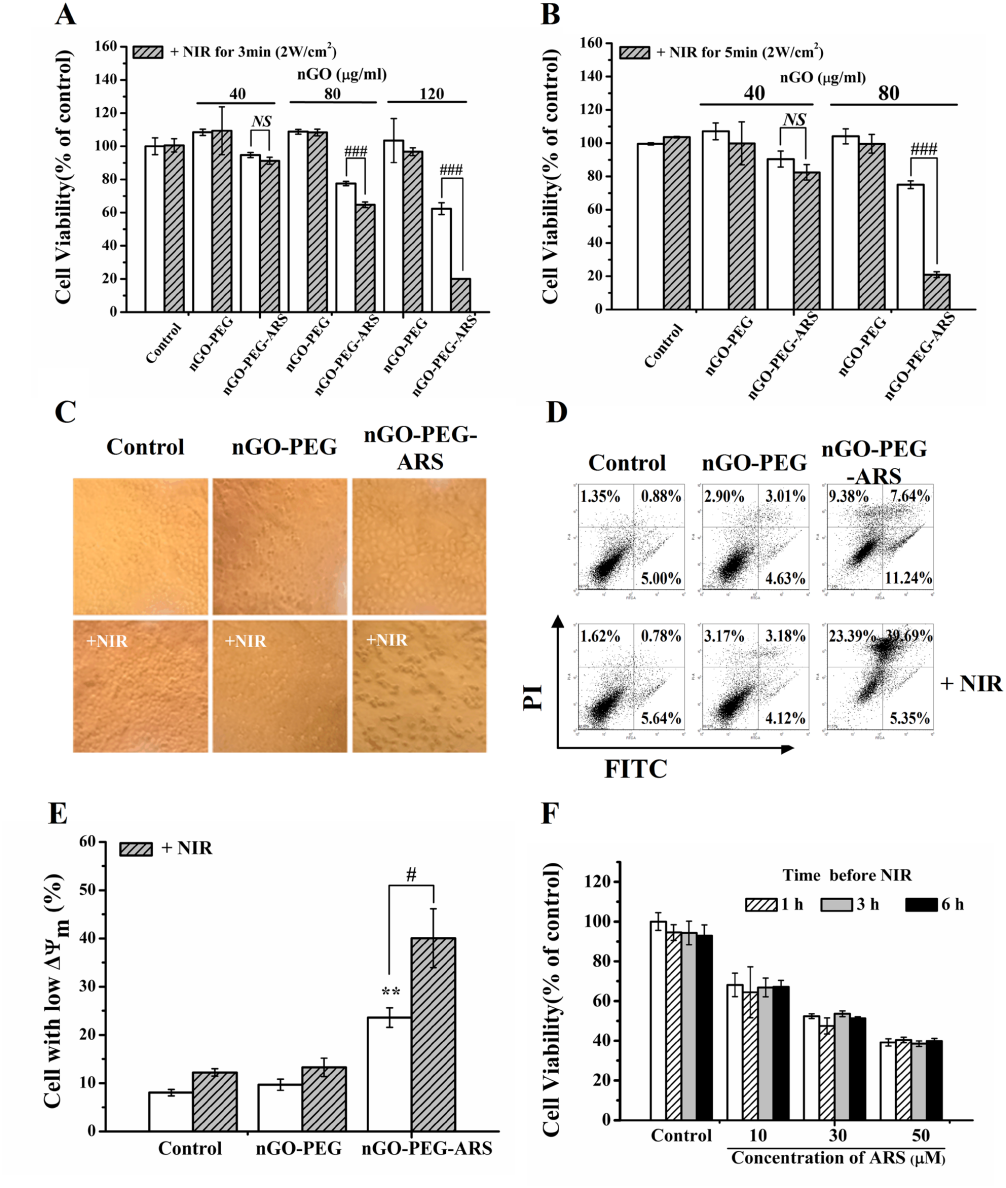
*In vitro* synergistic chemo-photothermal anti-cancer effect of nGO-PEG-ARS in HepG2 cells **(A** and **B)** Cytotoxicity of nGO-PEG and nGO-PEG-ARS with or without NIR irradiation (2 W/cm^2^) for 3 min (A) or 5 min (B) assessed by CCK-8 assay. ^**##**^*P* < 0.01 and ^**###**^*P* < 0.001; *NS*=no statistical significance. **(C)** Morphological analysis of cells treated with nothing, nGO-PEG andr nGO-PEG-ARS respectively for 48 h with or without NIR irradiation (5 W/cm^2^ for 15min). **(D)** Apoptosis induced by treatment with nGO-PEG or nGO-PEG-ARS for 48 h with or without NIR irradiation assessed by FCM analysis. After treatments, cells were stained with Annexin V-FITC/PI before FCM analysis. **(E)** FCM analysis on *ΔΨm* collapse induced by nGO-PEG or nGO-PEG-ARS for 48 h with or without NIR irradiation. After treatments, cells were stained with Rhodamine 123 before FCM analysis. ^**^P < 0.01, compared with control; ^***#***^*P* < 0.05. **(F)** Cytotoxicity of ARS (0, 30, 50 μM) with or without NIR irradiation at different time points (1, 3 and 6 h) assessed by CCK-8 assay. Those results represent duplicates with three independent experiments.

To investigate the synergistic chemo-photothermal anti-cancer effect of nGO-PEG-ARS, cells were incubated with different concentrations (containing 40-120 μg/mL of nGO) of nGO-PEG or nGO-PEG-ARS for 48 h in the presence or absence of NIR irradiation. The nGO-PEG-ARS group had an equivalent nGO dosage to the nGO-PEG group. Both the nGO-PEG group and nGO-PEG-ARS groups were irradiated by NIR light (2 W/cm^2^ for 3 min (Figure 3A) or 2 W/cm^2^ for 5 min (Figure 3B)). nGO-PEG group even at 120 μg/mL of concentration did not induce cytotoxicity (Figure 3A), indicating that the synthesized nGO-PEG is a relatively safe medicinal carrier and suitable for clinical and cell-biological applications. nGO-PEG-ARS (containing 40, 80 and 120 μg/mL of nGO) showed a dose-dependent cytotoxicity (Figure 3A), illustrating that the ARS loaded on nGO-PEG induced cell death. Moreover, although treatment with nGO-PEG and NIR irradiation did not induce cytotoxicity, NIR irradiation significantly enhanced the cytotoxicity of nGO-PEG-ARS (Figure 3A), indicating that NIR irradiation enhanced the chemotherapy effect of nGO-PEG-ARS. Especially, for the 120 μg/mL of nGO-PEG-ARS, NIR irradiation significantly enhanced the cytotoxicity of nGO-PEG-ARS from 37.60% (nGO-PEG-ARS alone) to 79.97% (Figure 3A), illustrating the remarkable synergistic chemo-photothermal effect of nGO-PEG-ARS in killing cancer cells. To further verify whether the photothermal effect of nGO-PEG enhanced the synergistic chemo-photothermal cytotoxicity of nGO-PEG-ARS, we prolonged NIR irradiation time from 3 min to 5 min, and found that irradiation of 2 W/cm^2^ laser for 5 min further enhanced the the cytotoxicity of ARS loaded on nGO-PEG (Figure 3B), further demonstrating the synergistic chemo-photothermal effect of nGO-PEG-ARS. Moreover, prolonging NIR irradiation time significantly enhanced the synergistic anti-cancer effect of nGO-PEG-ARS (Figure 3B), which may be due to the photothermally enhanced cell uptake amount of nGO-PEG-ARS. nGO-PEG/nGO-PEG-ARS containing 80 μg/mL of nGO and NIR irradiation of 2W/cm^2^ for 5 min were adopted in the following experiments without indicated condition.

Morphological analysis also showed that NIR irradiation significantly enhanced the nGO-PEG-ARS-induced cell death (Figure 3C). FCM analysis with Annexin V-FITC/PI double staining showed that NIR irradiation notably increased nGO-PEG-ARS-induced apoptosis with an increasing apoptotic rate from 18.88% (nGO-PEG-ARS group) to 45.04% n (GO-PEG-ARS plus NIR group) (Figure 3D), demonstrating the synergistic anti-cancer effect of nGO-PEG-ARS and NIR irradiation. Moreover, NIR irradiation also enhanced nGO-PEG-ARS-induced necrosis from 9.38% to 23.39%) (Figure 3D). We further performed FCM analysis with Rho123 staining, and found that nGO-PEG-ARS treatment for 48 h induced a significant *ΔΨm* loss which was significantly enhanced by NIR irradiation (Figure 3E), suggesting the involvement of mitochondria in the synergistic anti-cancer effect of nGO-PEG-ARS and NIR irradiation. Taken together, nGO-PEG-ARS exhibited an excellent synergistic chemo-photothermal anti-cancer effect.

To explore whether NIR irradiation directly interacted with ARS to enhance ARS-induced cell death, we assessed the cytotoxicity of various concentrations of ARS (10-50 μM) in the presence or absence of NIR irradiation. ARS induced a dose-dependent cytotoxicity, which was not enhanced by NIR irradiation (Figure 3F), suggesting that NIR irradiation did not directly enhance the cytotoxicity of ARS. Therefore, the remarkable synergistic chemo-photothermal anti-cancer effect of nGO-PEG-ARS was due to the photothermal effect of nGO-PEG and the chemotherapeutic effect of ARS.

### ONOO^─^ dominates the synergistic chemo-photothermal anti-cancer effect of nGO-PEG-ARS in HepG2 cells

**Figure 4:**
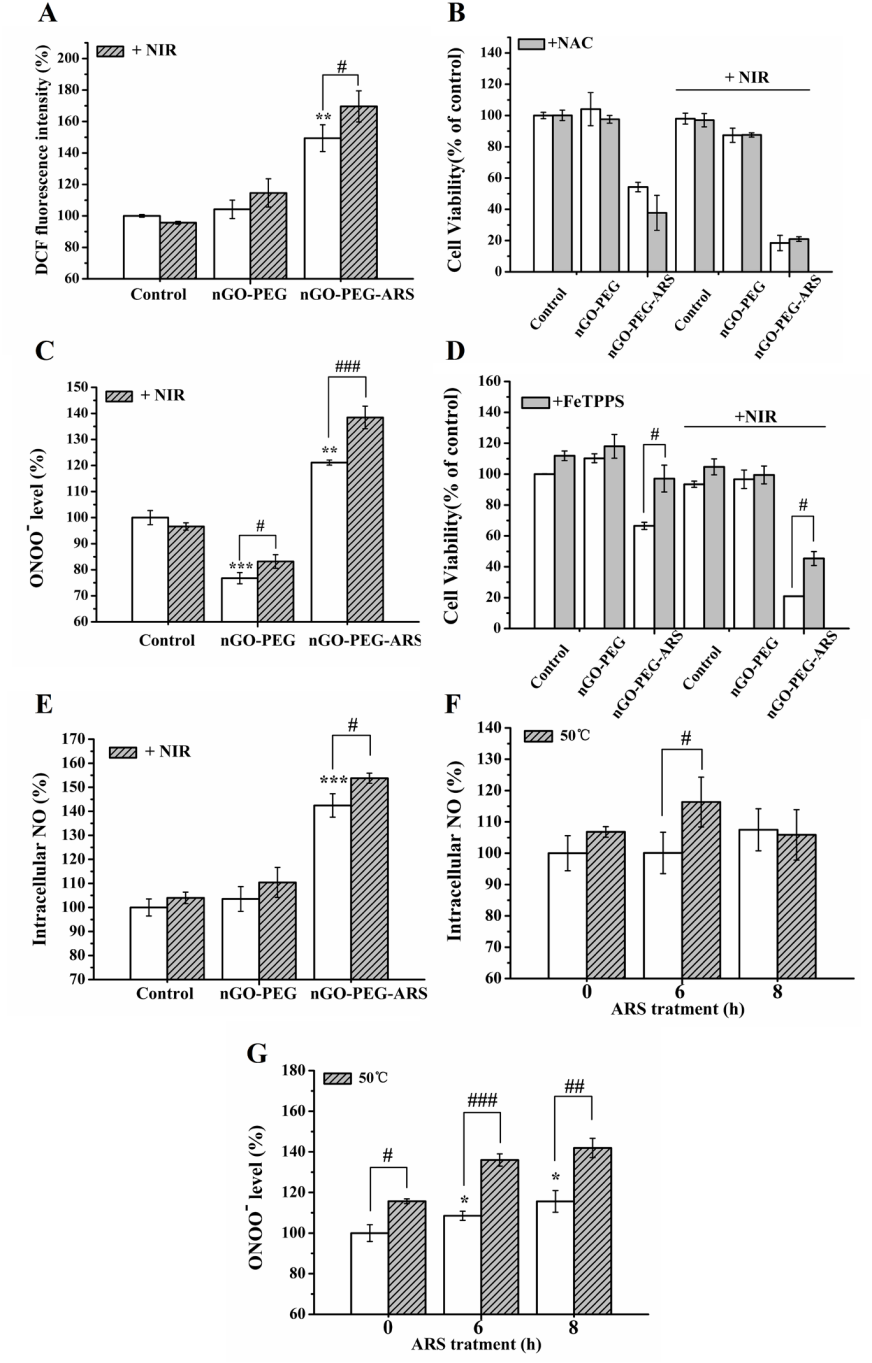
ONOO^-^ dominates the synergistic chemo-photothermal anti-cancer effect of nGO-PEG-ARS in HepG2 cells **(A)** ROS generation of cells treated with nGO-PEG and nGO-PEG-ARS respectively for 6 h with or without NIR irradiation (5 W/cm^2^ for 15 min) detected by FCM analysis. ^**^P < 0.01 compared with control; ^**#**^*P* < 0.05. **(B)** Effects of pretreatment with NAC on ROS generation induced by nGO-PEG or nGO-PEG-ARS for 48 h with or without NIR irradiation (5 W/cm^2^ for 15 min) before CCK-8 assay. **(C)** ONOO^—^ generation induced by nGO-PEG and nGO-PEG-ARS respectively for 6 h with or without NIR irradiation (5 W/cm^2^ for 15min) detected by FCM analysis. ^**^P < 0.01 and ^***^P < 0.01 compared with control; ^**#**^*P* < 0.05 and ^**##**^*P* < 0.01. **(D)** Effects of pretreatment with PeTTPS on ONOO^—^ generation induced by nGO-PEG ord nGO-PEG-ARS for 48 h with or without NIR irradiation before CCK-8 assay. ^**#**^*P* < 0.05. **(E)** NO generation by nGO-PEG or nGO-PEG-ARS with or without NIR irradiation detected by FCM analysis. ^***^P < 0.001 compared with control; ^**#**^*P* < 0.05. (F and G) NO generation **(F)** or ONOO^—^ generation **(G)** of cells treated with ARS for 0 h, 6 h and 8 h respectively in the presence or absence of heating cells to 50 °C (15 min) detected by FCM analysis. ^*^P < 0.05 compared with control; ^**#**^*P* < 0.05, ^**##**^*P* < 0.01 and ^**###**^*P* < 0.001. Those results represent duplicates with three independent experiments.

We recently demonstrated that ROS was not involved in ARS-induced apoptosis in HepG2 cells [48]. FCM analysis with DCF-DA staining showed that NIR irradiation significantly enhanced nGO-PEG-ARS-induced ROS generation (Figure 4A). However, CCK-8 assay showed that pretreatment with NAC (a ROS scavenger) did not inhibit the cytotoxicity of nGO-PEG-ARS treatment (Figure 4B), indicating that ROS did not mediate the chemotherapeutic effect of nGO-PEG-ARS. Moreover, NAC also did not inhibit the synergistic chemo-photothermal cytotoxicity of nGO-PEG-ARS plus NIR group (Figure 4B), suggesting that the synergistic chemo-photothermal anti-cancer effect of nGO-PEG-ARS was independent of ROS in HepG2 cells.

Based on our recent finding that ONOO^**─**^ from the reaction between NO and O2·^−^ dominated the sodium nitroprusside (SNP)-induced apoptosis in HepG2 cells [49], we speculated that ONOO^**─**^ might dominate the synergistic chemo-photothermal anti-cancer effect of nGO-PEG-ARS. As expected, FCM analysis with DHR 123 staining showed that nGO-PEG-ARS treatment for 6 h potently induced abundant ONOO^**─**^ generation which was significantly enhanced by NIR irradiation (Figure 4C). In addition, pretreatment with FeTTPS (a ONOO^**─**^ scavenger) for 2 h completely inhibited the cytotoxicity of nGO-PEG-ARS and significantly inhibited the cytotoxicity of nGO-PEG-ARS plus NIR irradiation (Figure 4D), illustrating that ONOO^**─**^ dominated the synergistic chemo-photothermal anti-cancer effect of nGO-PEG-ARS. Interestingly, nGO-PEG modestly enhanced cell viability (Figure 4D), which may be due to the declined ONOO^**─**^ generation in nGO-PEG group (Figure 3C). As ONOO^**─**^ is from the reaction between NO and O2·^−^ [50], we next evaluated the generation of NO in the synergistic effect of nGO-PEG-ARS and NIR irradiation, and found that nGO-PEG-ARS treatment for 6 h increased intracellular NO level which was significantly enhanced by NIR irradiation (Figure 4E), further verifying the key role of ONOO^**─**^ in the synergistic chemo-photothermal anti-cancer effect of nGO-PEG-ARS.

In fact, ARS induced time-dependent NO and ONOO^**─**^ generations, and ONOO^**─**^ generation occurred earlier than NO generation (Supplementary Figure 1), which may be in part due to the rapid consumption of NO to produce ONOO^**─**^ via the interaction of NO with O^2^·^−^ during NO generation. Therefore, the abundant NO and ONOO^**─**^ generations in nGO-PEG-ARS group (Figure 4C and 4E) might be induced by the ARS loaded on nGO-PEG. Compared with control, NIR irradiation did decrease the ONOO^**─**^ generation in nGO-PEG groups, while significantly increased the ONOO^**─**^ generation of nGO-PEG-ARS group (Figures 4C and 4E), indicating that the photothermal effect of nGO-PEG under NIR irradiation triggered ARS loaded nGO-PEG-ARS to produced more ONOO^**─**^. To further verify whether the photothermal effect of nGO-PEG by NIR irradiation enhanced ARS-induced NO and ONOO^**─**^ generations, cells treated with ARS for various time (0 h, 6 h and 8 h) were cultured at 50 °C for 15 min before FCM analysis, exhibiting that 50 °C significantly enhanced ARS-induced NO (Figure 4F) and ONOO^**─**^ (Figure 4G) generations. Combining our previous findings that nGO-PEG plus NIR irradiation did not induce cytotoxicity (Figure 3A) and NIR irradiation neither induced ONOO^**─**^ generation nor triggered nGO-PEG to induce ONOO^**─**^ generation (Figure 4C), it is reasonable to infer that the localized photothermal effect of nGO-PEG by NIR irradiation triggers ARS loaded on nGO-PEG to produce more NO and ONOO^**─**^ to mediate the synergistic chemo-photothermal anti-cancer effect of nGO-PEG-ARS.

### *In vivo* synergistic chemo-photothermal anti-cancer effect of nGO-PEG-ARS

**Figure 5:**
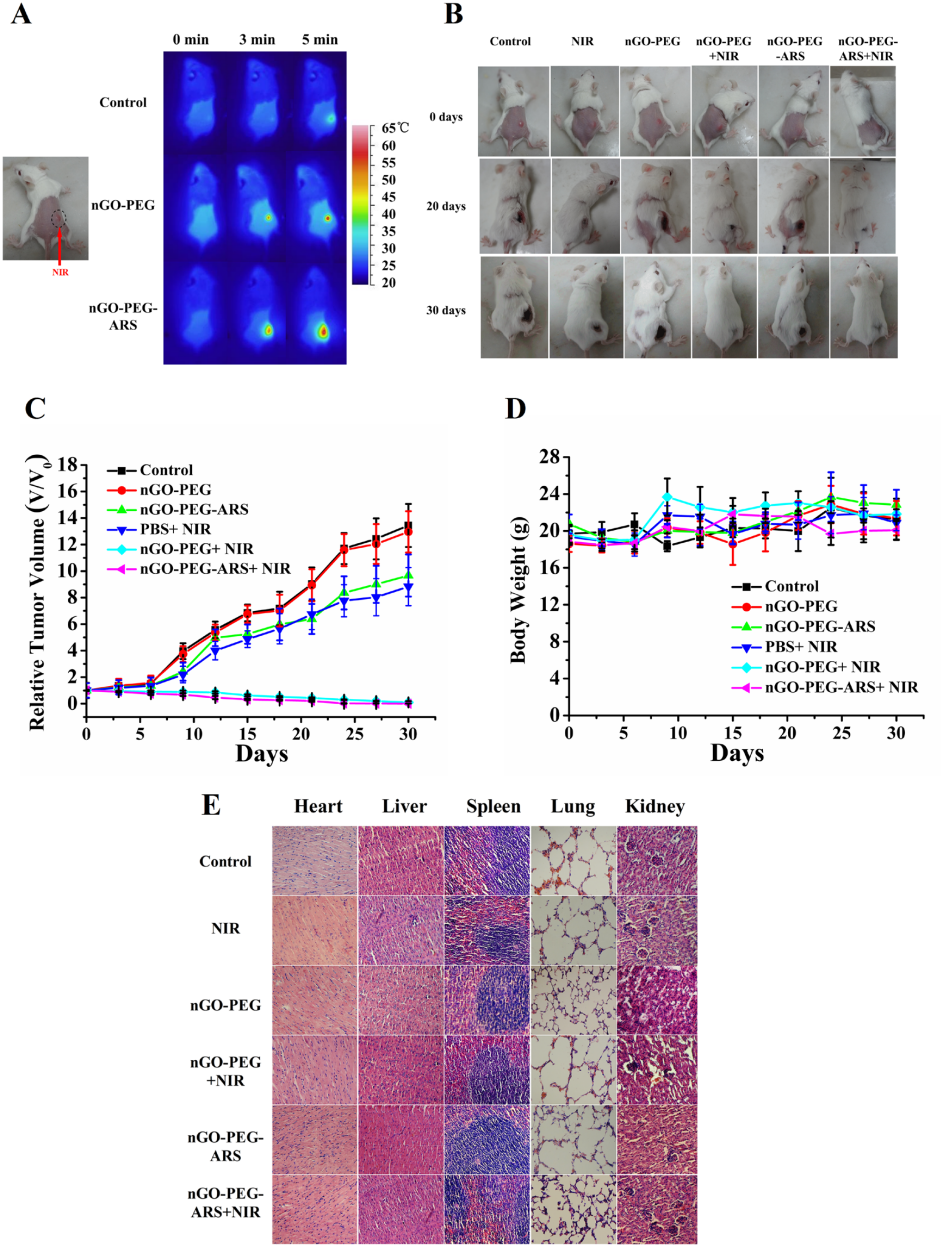
*In vivo* synergistic chemo-photothermal anti-cancer effect of nGO-PEG-ARS **(A)** Infrared thermal images of tumor-bearing mice with six treatments (control, nGO-PEG, nGO-PEG-ARS, NIR, nGO-PEG+NIR and nGO-PEG-ARS+NIR). Before Infrared thermal imaging, the mice were fastened by adhesive tape and back hair was removed with depilatory cream. **(B)** Photographs of representative mice before and after different treatment (30 days). Tumors were enclosed by the black dotted lines. **(C)** Quantitative analysis on the effects of six treatments on tumor size. **(D)** Changes with time in body weight achieved from mice injected six treatments. Values are means ± SD (n = 4). **(E)** Representative H&E stained images of major organs including heart, lung, spleen, kidney and liver collected from mice.

To investigate the *in vivo* synergistic anti-cancer efficacy of nGO-PEG-ARS, comparative efficacy studies on tumor-inhibiting effectiveness were conducted. Mice bearing 4T1 tumors were distributed into five groups and were treated according to protocols as summarized in “Materials and methods”. The temperature of the tumor region in the mice was monitored using an infrared thermal camera. Upon 808 nm laser irradiation within 5 min, the surface temperature of the tumors region in the nGO-PEG- and nGO-PEG-ARS-injected mice quickly reached over 50 °C (Figure 5A) which was sufficient for PTT [51]. In contrast, the surface temperatures of control group injected with PBS only reached 40 °C after NIR irradiation (Figure 5A). In the next 30 days after NIR irradiation, the changes of relative tumor sizes and weights were monitored every three days, and as a function of time were plotted after treatment (Figure 5C) and photographs of the test mice (before, and on day 20, and day 30) were showed in Figure 5B. As shown in Figure 5C, the tumors of the mice in the nGO-PEG group grew rapidly and had a similar size to control group, while for mice in nGO-PEG-ARS group and nGO-PEG plus NIR group, the tumor was inhibited to a certain extent in the early 6 days, but began to grow rapidly afterwards. The tumor was completely ablated within 15 days in nGO-PEG-ARS plus NIR group, while in nGO-PEG plus NIR group, the tumor was completely ablated within 21 days (Figure 5C), demonstrating the combinational therapy group enhanced anti-tumor effect. Moreover, the tumors in both nGO-PEG plus NIR and nGO-PEG-ARS plus NIR groups did not increase within 30 days (Figures 5B and 5C), suggesting that the photothermal effect of nGO-PEG is suitable for PTT *in vivo*. These results illustrated that nGO-PEG-ARS combined with NIR irradiation could realize an efficient synergistic chemo-photothermal therapy effect for antitumor therapy.

Furthermore, body weight is an important parameter to evaluate the systemic toxicity of a material to the body. As given in Figure 5D, the body weight of the six groups had no obvious decrease with prolonged time, implying that treatment with nGO-PEG-ARS plus NIR had little adverse side effects. Subsequently, we carried histological analysis to evaluate the potential side effects of nGO-PEG-ARS on major organs (heart, liver, spleen, lung and kidney) of tumor bearing mice. H&E staining histological sections showed no apparent histological lesion or any other tissue damage for the mice treated with nGO-PEG-ARS in the presence or absence of NIR for 30 days (Figure 5E), indicating the safe application of nGO-PEG-ARS *in vivo*.

## MATERIALS AND METHODS

### Materials

GO powder was purchased from XF NANO Co., Ltd. (Nanjing, China). N-hydroxysuc-cinimide (NHS) and N-(3-(dimethylamino)propyl-N′-ethylcarbodiimide) hydrochloride (EDC) were purchased from Sigma-Aldrich (Shanghai, China). 8-Armpolyethylene glycol-amine (10 kDa, PEG-NH2) was purchased from Seebio Biotech Inc. (Shanghai, China). ARS was obtained from Chongqing Holley Pharmaceutical Co., Ltd. (Chongqing, China). Fetal bovine serum (FBS) was purchased from Sijiqing (Zhejiang, China). Dulbecco’s modified Eagle’s medium (DMEM) was purchased from Thermo Scientific (USA). Roswell Park Memorial Institute 1640 (RPMI 1640) was purchased from Gibco (Grand Island).

### Synthesis of nGO-PEG-ARS

GO was obtained by oxidation of graphite following the modified Hummers method [52]. Nanosized GO (nGO) was obtained by ultrasonication of GO and converted to carboxylated nGO (nGO −COOH). As shown in Scheme 1, nGO-COOH was conjugated with amine-terminated 8-armed PEG (PEG- NH_2_) (10 kDa) via amido bond using EDC chemistry for 12 h, and the PEGylated nGO (PEG−nGO, GP) was then subsequently covalently functionalized with ARS by EDC/NHS activation reaction to synthetize nGO-PEG-ARS.

### Characterization of nGO-PEG-ARS

The sizes and zeta potentials of nGO, nGO-PEG and nGO-PEG-ARS were confirmed by Zetasizer Nano ZS (Malvern Instruments, Malvern, UK). UV-vis spectra were performed by a UV-vis spectrometer (Lambda 35, PerkineElmer, USA) with a 1 cm quartz cuvette. Fourier transfer infrared (FT-IR) spectra were recorded on a Nicolet 6700 FT-IR spectrometer (Thermo Fisher Scientific, USA). Thermogravimetric analyses (TGA) were performed on a thermogravimetric analyzer (NETZSCH/TG209, Germany) from room temperature to 700 °C at a heating rate of 5 °C min^-1^ under N_2_ flow. The images of all samples (nGO, nGO-PEG and nGO-PEG-ARS with the same dose of 10 μg/mL nGO) were recorded using a digital camera (Sony, Tokyo, Japan).

### Cell uptake of nGO-PEG-ARS

The nGO-PEG-ARS was labeled by fluorescein isothiocyanate (FITC, Sigma). In brief, the solution of nGO-PEG-ARS (approximately 0.5 mg/mL, 1.0 mL) was mixed with 0.1 mL FITC (13 mM) dissolved in DMSO and then stirred over night at room temperature. The resulting mixtures were filtrated through 30 kDa filters Millipore and centrifuged at 6500 × g to remove aggregated FITC, giving water-solubility FITC/nGO-PEG-ARS. The whole procedures were operated in dark place. HepG2 cells were plated into 6-well plates at a density of 1 × 10^5^ cells/well and incubated with 80 μg/mL (final concentration) of FITC/nGO-PEG-ARS for 2, 4 and 8 h with or without NIR irradiation respectively in 1.0 mL DMEM medium containing 10% fetal bovine serum, and then, the cells were rinsed and collected by phosphate buffered saline (PBS). The uptake ratio of nGO-PEG-ARS by HepG2 cells was measured by flow cytometry (FCM, FACSCantoII, Becton Drive, New Jersey, USA) using FITC labeled on nGO-PEG-ARS, and 10,000 events were recorded for each FCM analysis.

### Evaluation of photothermal performance

The test solution (1 mL) was introduced in an eppendorf tube (1.5 mL) and exposed to the 808 nm laser irradiation at a power density of 2 W/cm^2^ for 5 min. The temperatures of water, nGO, nGO-PEG and nGO-PEG-ARS solutions were measured by a thermocouple thermometer (Fluke 51II, Lake Mary, FL, USA) every other 30 s. In addition, the thermal images of these solutions were also measured by an infrared thermal camera (TVS200EX, NEC, Minato, Tokyo, Japan). All experiments were conducted at room temperature.

### Cell viability measurement

HepG2 cells were cultured in a DMEM medium containing 10% fetal bovine serum at 37 °C in a humidified 5% CO2 atmosphere. For cell toxicity assay, HepG2 cells were plated into 96-well plates at a density of 1 × 10^4^ cells/well with 100 μL of medium and incubated for 24 h. After removing DMEM medium, the cells were exposed to 40, 80 and 120 μg/mL of nGO-PEG or nGO-PEG-ARS respectively for 4 h in 100 μL of media containing 10% fetal bovine serum, and then irradiated with 2 W/cm^2^ of 808 nm NIR laser for 3 min or 2 W/cm^2^ of 808 nm NIR laser for 5 min followed by further incubation for 44 h. Cell viability was evaluated by using Cell Counting Kit-8 (CCK-8, Dojindo, Japan) assay as described previously [53]. Results reflect the average of at least three replicates.

Cell apoptosis was quantified by FCM analysis using Annexin V-FITC/PI apoptosis detection kit (Bestbio, Shanghai, China) according to the manufacture's protocol as previously described [53]. Apoptotic cells were those stained with Annexin V-FITC^+^/PI^−^(early apoptotic cells) and Annexin V-FITC^+^/PI^+^ (late apoptotic cells).

### Measurement of mitochondrial membrane potential (ΔΨm)

Characteristic breakdown of mitochondrial transmemberane potential (*ΔΨm*) was analyzed by FCM assay using *ΔΨm*-specific stain Rho123 as previously described [53]. Briefly, cells were harvested and stained with 5 μM Rho123 at 37°C for 20 min in the dark, and then washed with PBS twice and subsequently assayed by FCM. Proportion of cells with low Rho123 fluorescence indicated the loss of *ΔΨm*.

### Measurement of ROS and peroxynitrite (ONOO^─^)

ROS and ONOO^**─**^ generations inside living cells were measured by FCM with an oxidation-sensitive probe 2′, 7′-dichlorodihydrofluorescein diacetate (DCF-DA, Sigma, USA) and Dihydrorhodamine 123 (DHR 123, Wuhan, Aimeijie) as described previously [49]. DCF-DA is cleaved by nonspecific esterases and becomes highly fluorescent DCF upon oxidation by ROS. DHR 123 is oxidized by ONOO^**─**^ to the highly fluorescent product rhodamine. HepG2 was cultured with nGO-PEG and nGO-PEG-ARS for the indicated times with or without 808 nm NIR laser irradiation (5 W/cm^2^) for 15min, and then the cells were stained with 20 μM DCF-DA in PBS for 30 min or with 10 μM DHR 123 for 20 min in the dark at 37 °C. Then the cells were harvested and washed twice with PBS before FCM analysis.

### Measurement of nitric oxide (NO)

Intracellular NO level was quantified by using FCM analysis with DAF-FM DA staining as described previously [49]. HepG2 cells were cultured with nGO-PEG and nGO-PEG-ARS for the time indicated with or without 5 W/cm^2^ of 808 nm NIR laser for 15min, and then the cells were stained with 5 μM DAF-FM DA for 30 min at 37 °C. After washing with PBS three times, the samples were analyzed by FCM analysis.

### *In vivo* antitumor effect and systemic toxicity

Female Balb/c mice (4-6 weeks old) were obtained from Laboratory animal center of Sun Yet-sen University and approved by Guangdong Province Experimental Animals Monitoring Institute. 4T1 tumor bearing mice were established by subcutaneous injection of 5 × 10^6^ cells in 200 mL PBS into the flank region of Balb/c mice. The dimension of tumors was monitored by digital calipers. After tumor size reached approximately 60 mm^3^, the mice were randomized into 6 different treatment groups (5 mice per group): control group, nGO-PEG group, nGO-PEG-ARS group, NIR group, nGO-PEG plus NIR group and nGO-PEG-ARS plus NIR group. Balb/c mice bearing 4T1 tumors in different groups were intratumorally injected with nGO-PEG, nGO-PEG-ARS (an equivalent nGO dosage of 10 mg/kg), respectively. For NIR irradiation groups, after 2 h injection, the tumor region of mice was irradiated by 808 nm NIR laser for 15 min. During NIR irradiation, full-body thermographic images were captured using an infrared camera (NEC, Japan). After treatment, all mice were returned to animal housing and tumor volumes were tracked every 3 days by digital caliper measurements. The tumor volume was calculated by formula V = (longest dimension) ∙ (shortest dimension)^2^/2. Control and nanocomposites-treated mice were sacrificed 30 days after treatment. Major organs of those mice were collected, fixed in 4% formalin, conducted with paraffin embedded sections, stained with hematoxylin and eosin (H&E), and examined under a digital microscope.

## CONCLUSION

In summary, we successfully developed an ARS-loaded nGO-PEG composite (NGO-PEG-ARS) by covalently linking ARS to PEGylated nGO for synergistic chemo-photothermal therapy of cancer. nGO-PEG-ARS exhibited excellent dispersibility in physiological environments and synergistic chemo-photothermal anti-cancer effect *in vitro* and *in vivo*. ONOO^**─**^ dominated the synergistic chemo-photothermal anti-cancer effect of nGO-PEG-ARS. The photothermal effect of nGO-PEG under NIR irradiation significantly enhanced not only cell uptake but also ONOO^**─**^ generation of nGO-PEG-ARS, contributing to the outstanding synergistic chemo-photothermal anti-cancer ability of nGO-PEG-ARS.

## SUPPLEMENTARY MATERIALS FIGURE



## Abbreviations

ARS: Artesunate
CCK-8: Cell Counting Kit-8
DCF-DA: 2′, 7′-dichlorodihydrofluorescein diacetate
DHA: Dihydroartemisin
DHR 123: Dihydrorhodamine 123
DMEM: Dulbecco’s modified Eagle’s medium
DOX: Doxorubicin
EDC: N-(3-(dimethylamino)propyl-N′-ethylcarbodiimide) hydrochloride
FBS: Fetal bovine serum
FCM: flow cytometry
FeTTPS: Fe(III) 5,10,15,20-tetrakis (4-sulfonatophenyl) porphyrinato chloride
FITC: Fluorescein isothiocyanate
FT-IR: Fourier transfer infrared
NAC: N-Acetyle-Cysteine
nGO: Nano-graphene oxide
NHS: N-hydroxysuc-cinimide
NIR: Near-infrared laser
NO: Nitric oxide
ONOO^─^: Peroxynitrite
PBS: Phosphate buffered saline
PDT: photodynamic therapy
PEG: Polyethylene glycol
PTT: Photothermal therapy
ROS: Reactive oxygen species
RPMI 1640: Roswell Park Memorial Institute 1640
RV: Resveratrol
SNP: Sodium nitroprusside
TGA: Thermogravimetric analyses
*ΔΨm*: Mitochondrial transmemberane potential
