# Supplementary material for: Artesunate-modified nano-graphene oxide for chemo-photothermal cancer therapy
Source: Oncotarget. 2017 Sep 23;8(55):93800–12. doi: 10.18632/oncotarget.21191 (PMC5706836; doi:10.18632/oncotarget.21191)
Supplement: Supplementary file 1 [file oncotarget-08-93800-s001.pdf]

Artesunate-modified nano-graphene oxide for chemo-photothermal cancer therapy

SUPPLEMENTARY MATERIALS

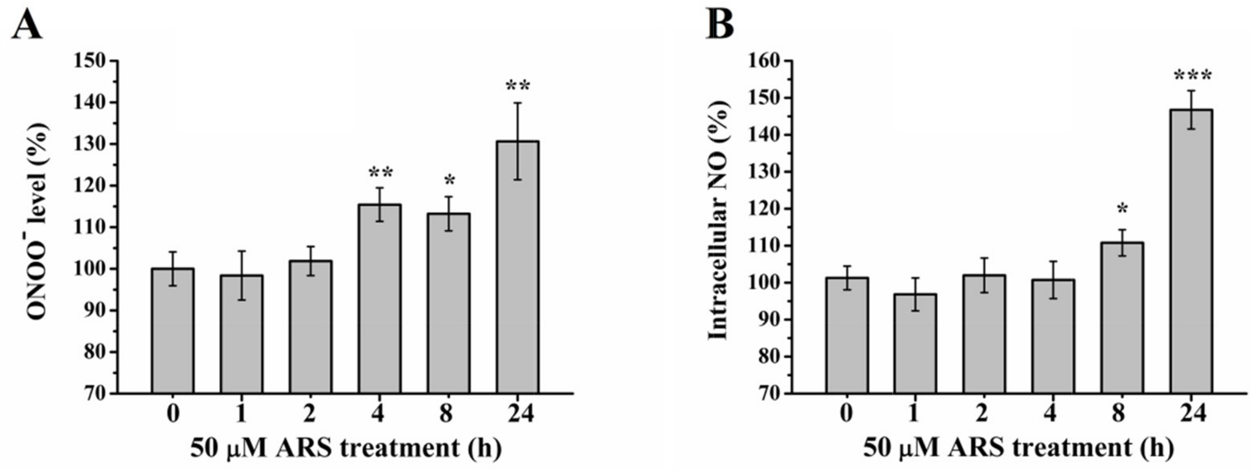

Supplementary Figure 1: ARS induces time-dependent ONOO─ and NO generation. (**A** and **B**) $ONOO^-$ generation (A) and NO generation (B) induced by 50 μM ARS detected by FCM analysis. $^{*}P < 0.05$, $^{**}P < 0.01$ and $^{***}P < 0.001$, compared with control (0 h).
